# Lower limb deformity following proximal tibia physeal injury: long-term follow-up

**DOI:** 10.1007/s10195-012-0179-x

**Published:** 2012-02-11

**Authors:** Aristides N. Vrettakos, Dimosthenis C. Evaggelidis, Margaritis J. Kyrkos, Apostolos V. Tsatsos, Alexandros Nenopoulos, Theodoros Beslikas

**Affiliations:** 1 2nd Orthopaedic Department, Aristotle University of Thessaloniki, Thessaloniki, Greece; 2 Orthopaedic Clinic of “Panagia” Department, “Agios Pavlos” Hospital, N. Plastira 22, 55132 Thessaloniki, Greece

**Keywords:** Proximal tibial physeal injury, Length disturbance, Axial disturbance, Pediatric fractures

## Abstract

**Background:**

Proximal tibial physeal injuries are quite rare, but their complications can be of great importance. The purpose of this study was to evaluate the effect of this injury on the axis and length of a child’s limb.

**Materials and methods:**

This study focused on 12 patients with proximal physeal injury of the tibia (8 boys and 4 girls; mean age at the time of injury: 8.9 years). Injuries were classified according to the Salter–Harris scheme into 5 types (type II—7 patients, type III—3 patients, type IV—1 patient, type V—1 patient). In 5 cases, a coexisting fracture of the injured limb was observed (fibular fracture—3 cases, intercondylar fracture—1 case, tibial tubercle fracture—1 case). Ten patients were treated conservatively and 2 patients underwent an operation. Seven of the 12 patients were available for long-term follow-up, with a mean duration of 14.4 years (11.2–22.0 years).

**Results:**

Angular deformity was observed in 6 of the 7 patients, with a mean valgus deformity of 2.7°, within an average of 5.8 months after the injury. After 3 years of follow-up, complete remodeling was observed in all of those 6 cases (4 of the patients were treated conservatively and 2 underwent surgery). One patient developed 6 mm of tibial shortening. No functional limitation or pain was recorded in any of the patients during the follow-up.

**Conclusions:**

Injury to the proximal tibial epiphysis, while rare, may result in angular or length disturbance, regardless of the initial treatment (conservative or surgical). Parents should always be informed of this possibility, and long follow-up is indicated. Nevertheless, this type of injury rarely results in functional limitations.

## Introduction

Proximal physeal injuries of the tibia are quite rare, since they constitute only 0.6% of the fractures of the long bones in children [1]. The proximal tibial epiphysis is protected by the contralateral knee and the surrounding soft tissues (fibular head ligaments, patellar tendon, insertion of semitendinosus and medial collateral ligaments into the proximal metaphysis) [1–3]. Nevertheless, complications of these injuries are of great importance [4], and therefore require exceptional attention, especially when the injury is initially diagnosed. Proximal tibial physeal injury may result when the forces applied to the limb produce a moment of hyperextension and varus or valgus alignment, with fracture of the physeal plate. In those cases, a disturbance of the tibial axis or length may be observed [3–6]. Also, due to its proximity, the popliteal artery may be injured when the tibial shaft is posteriorly displaced [2]. These fractures can be treated either conservatively or surgically, depending on the injury type, the reduction quality, and the post-reduction stability of the fracture fragment. The study described in this paper focused on how proximal tibial physeal injuries can affect the length of the tibia and the alignment of its axis.

## Materials and methods

Over a period of 22 years (from 1984 to 2006), 12 children with proximal tibial physeal injury were treated in our department. They were 8 boys and 4 girls, with a mean age of 8.9 years (range: 3–13 years). The right leg was injured in 7 cases and the left in 5. Seven children had a fall, 3 were injured while participating in sports, and 2 had sustained the injury in a road traffic accident. Isolated avulsion fractures of the tibial tubercle or the intercondylar eminence were not included in this study.

Regarding the type of physeal injury, there were 7 Salter–Harris (S-H) type II fractures, 3 S-H type III fractures, 1 S-H type IV fracture, and 1 S-H type V fracture. One of the patients had a concomitant tibial tubercle fracture, and another patient had an intercondylar eminence fracture. In 3 cases there was also a fibula fracture.

Ten cases were treated conservatively. Four patients with an undisplaced fracture were treated with a long leg cast (retaining 30° of knee flexion), which was removed after 6–8 weeks (depending on the child’s age). Treatment was completed with mobilization and gradual weight-bearing. For 5 cases in whom the displacement was over 2 mm, the fractures were treated with closed reduction under sedation or general anesthesia. Distal neurovascular function was assessed before and after the reduction and, since the fracture was stable with the knee flexed and the distal circulation was not compromised, a long leg cast with 60° of knee flexion was placed for 4 weeks. Subsequently, a new long leg cast with 30° of knee flexion was applied for 2 weeks more. After the cast was removed, a mobilization program was followed, and the patients were gradually ambulated. In 1 case of a S-H type III injury with a coexisting fracture of the intercondylar eminence, a closed reduction was performed and a long leg cast was placed, with the knee fully extended. After 4 weeks, this cast was replaced with a long leg cast with 30° of knee flexion, which was removed after 2 more weeks.

Surgical treatment (open reduction and internal fixation) had to be performed in two cases. The first case was a S-H type III fracture with a coexisting severely displaced fracture of the tibial tubercle, which was treated with open reduction and internal fixation with a cannulated screw. The other case was a neglected S-H type V physeal fracture, with established varus deformity and consequent tibial shortening. The patient presented 1 year after the injury and a corrective osteotomy was performed.

Patients were initially re-evaluated at 1, 2, and 6 weeks and at 3, 6, 12, and 24 months. Seven of the 12 patients were available for long-term follow-up, with a mean duration of 14.4 years (11.2–22.0 years). They underwent clinical and radiological evaluations, taking plain radiographs of the tibia (anteroposterior and lateral). Radiographic assessment was performed with the patients in the supine position, since weight-bearing was restricted for 6–8 weeks, and because of their young age and poor cooperation. Radiological evaluation included measurement of the lengths of the tibia and fibula, and measurement of the tibial metaphyseal–diaphyseal angle on the affected side in comparison to the normal limb, according to Levine and Drennan [7].

This was a retrospective cohort study, conducted after all of the patients and their parents had given their informed consent. The study was authorized by the Aristotle University Ethics Committee, and was performed in accordance with the ethical standards of the 1964 Declaration of Helsinki, as revised in 2000.

## Results

Seven of the 12 patients were available for long-term follow-up, with a mean duration of 14.4 years (11.2–22 years). Clinical examination revealed normal ranges of motion in hips, knees, and ankle joints in all children. There was no pain or other functional limitation in any joint. Results are summarized in Table 1.

**Table 1:** Data on the 7 patients that were available for long-term follow-up

Injury type (Salter–Harris classification)	Patient	Concurrent injuries	Treatment	Initial deformity (4–6 months post-injury)		Mid-term deformity (2.5–3 years post-injury)		Final follow-up (years)^a^
				Axial disturbance	Length disturbance	Axial disturbance	Length disturbance	
II	1	None	Conservative	0°	0 mm	0°	0 mm	13.8
	2	None	Conservative	4° valgus	0 mm	0°	0 mm	22.0
	3	None	Conservative	2° valgus	0 mm	0°	0 mm	15.0
	4	None	Conservative	3° varus	0 mm	0°	0 mm	12.6
III	5	Tibial tubercle fracture	Operative	3° varus	0 mm	0°	0 mm	13.5
	6	Intercondylar eminence fracture	Conservative and operative^b^	8° varus	0 mm	0°	0 mm	12.9
V^c^	7	None	Operative, 1 year post-injury	8.5° varus	6 mm shortening	2° varus	0 mm^d^	11.2

^a^No difference was observed in any patient in terms of axial and length deformity between the mid-term and final evaluation. ^b^ The operation was performed 1 year post-injury, after the varus injury had developed. ^c^ The patient did not receive treatment initially; he presented 1 year post-injury with an established deformity and he was operated on. ^d^ Measurement was performed 4 years post-operatively

**Fig. 1:**
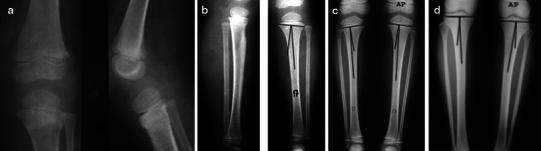
**a** Anteroposterior and lateral radiographs of a 5 year old boy with a Salter–Harris type II proximal physeal injury of the left tibia. **b** One year post-injury, with a valgus deformity of 4° compared to the normal side. **c** Three years post-injury; almost complete remodeling is observed. **d** Twenty-two years post-injury, without any angular deformity

**Fig. 2:**
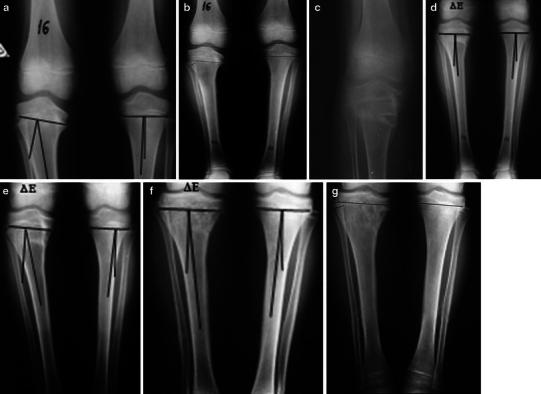
**a** Anteroposterior radiographs of a 13 year old boy with an S-H type V proximal physeal injury of the right tibia, 12 months post-injury. **b** Anteroposterior radiographs of the same patient 12 months post-injury, showing a tibial length discrepancy of 6 mm. **c** The patient underwent a correctional osteotomy. **d** Five months after the correctional osteotomy, without angular deformity. **e** Two years after the correctional osteotomy. Malalignment of the tibial axis is present. **f** Four years after the correctional osteotomy. Despite the partial remodeling, a residual valgus deformity of 2° can be observed. **g** Four years after the correctional osteotomy. No tibial or fibular length discrepancy is apparent

The limb axis measurements for the various fracture types were as follows: concerning the 4 patients with a S-H type II injury, 1 of them had no angle deformity, 2 of them had valgus deformities of 4° (Fig. 1) and 2°, respectively, and the other had a varus deformity of 3°. All of these axial deviations were self-corrected during follow-up. One of the 2 patients with an S-H type III fracture had a coexisting fracture of the tibial tubercle and developed a varus deformity of 3°. Remodeling was observed in a later evaluation. The other patient with an S-H type III injury had a coexisting fracture of the intercondylar eminence. He was initially treated conservatively, but a varus deformity of 8° developed and he underwent a corrective valgus osteotomy 1 year post-traumatically. The axis corrected itself without any recurrence of deformity. The patient with the S-H type V fracture presented with an established varus deformity of 8.5° 1 year post-traumatically, and he was treated with a corrective valgus osteotomy (Fig. 2). The deformity relapsed within the next 2 years and was subsequently partially corrected within the next 2 years, finally remaining at 2° in varus. No angular deformity was observed in 1 of the 7 patients, while the remaining 6 patients presented with axial deviations. Four of them had a varus deformity with a mean of 5.6°, and 2 patients had a valgus deformity with a mean of 3°. The angular deformity was observed at a mean 5.8 months after the injury. In 4 of the 6 patients, the axial deviation self-corrected within 3 years, while the remaining two underwent surgery and also had no axial deformity at the 3-year evaluation. No further alteration in axial deformity was observed in any patient between the 3-year evaluation and their longest follow-up.

Regarding the tibial length, 1 patient with a S-H type V physeal injury presented with tibial shortening of 6 mm. He underwent corrective osteotomy for his concurrent axial deformity, and no tibial length disturbance was recorded after 4 years of follow-up. No tibial length distortion was observed in the remaining 6 patients.

## Discussion

Proximal physeal injury of the tibia is very rare, but complications of such injuries can be serious, including ligamentous injuries, vascular complications, compartment syndrome, knee instability, osteoarthritis, tibial shortening, and axial deformity [8–10].

Concerning growth disturbance, Gautier et al. [10], in a meta-analysis of published series including 110 patients, mention that 25% had posttraumatic growth deformities of more than 25 mm in length or more than 5° of angulation. Another 21% had deformities of less than 25 mm of length or less than 5° of angulation. Thus, the total rate of deformity was over 45%. In our group of 7 patients, 1 presented tibial shortening of 6 mm and 6 presented angular deformity. This high percentage of angular deformity in our group of patients may be explained by their young age at the time of injury (mean 8.9 years old), so they were not near physeal closure. Considering that this axial distortion was observed at an average of 5.8 months post-injury, our belief is that the initial angular deformity is a consequence of the asymmetrical growth caused by the physeal disturbance. Ogden [11] suggests that the diminished blood flow impedes cellular replacement in the hypertrophic cell zone of the epiphyseal plate. On the contrary, the zone of proliferating cartilage with intact blood flow continues to grow, resulting in physeal plate thickening. Although valgus angulation is reported to be the most frequent angular deformity [10], varus angulation was more common in our group of patients. This may be explained by remarks made by Kessel [12], who suggests that injury on the medial side of the proximal tibial physeal plate may cause an interruption of the tibial growth, in contrast to normal fibular development, resulting in a tibial varus deformity. It is also noted that angular deformities after significant physeal plate injuries (apart from tibial length disturbances) may also result in tibial rotation. This is due to the different levels of superior and inferior tibiofibular articulation, which results in a 6° angle between them. As noted by Gautier et al. [10], the growth deformity is occasionally an overgrowth in the involved leg, despite the described damage to the physis. This phenomenon is thought to be secondary to indiscriminate stimulation of all physeal plates of the extremity due to increased blood perfusion, with consequent leg length discrepancy [13]. Long-term follow-up is essential for angular deformity, since it may fully or partially self-correct or relapse after a corrective osteotomy. This depends on the severity of the injury to chondrocytes in the physeal plate, which may not be radiologically identified and is retroactively diagnosed. This finding of our study is in agreement with Burkhart [15].

Knee osteoarthritis can be another serious complication, as noted by Pournaras [4], Poulsen et al. [8], and Bertin and Goble [14]. No osteoarthritic lesions were observed in the early or late radiological evaluations of our group, probably because of the non-intra-articular injury type of most of our patients, and because of the complete remodeling that was observed within 3 years post-injury.

Burkhart and Peterson [15], as well as Gautier [10], point out that the Salter–Harris classification may not be reliable for obtaining prognostic information about the rate and extent of posttraumatic deformities following proximal tibial physeal injuries. This may also be supported by the study of Shelton and Canale [16]. It is noteworthy that 3 out of 4 of our patients with an S-H type II injury developed an angular deformity.

The main weakness of this study is the small number of patients included (12 patients, 7 available for long-term follow-up), due to the rarity of this injury. The main strength of this study is the long-term follow-up (average of 14.4 years, range 11.2–22 years).

Overall, we can conclude that proximal physeal injury of the tibia may result in angular or length disturbance, regardless of the initial treatment (conservative or surgical). Parents should always be informed of this possibility, and long follow-up is indicated. Nevertheless, this type of injury rarely results in functional limitations.
